# Regulatory System for Stem/Progenitor Cell Niches in the Adult Rodent Pituitary

**DOI:** 10.3390/ijms17010075

**Published:** 2016-01-09

**Authors:** Saishu Yoshida, Takako Kato, Yukio Kato

**Affiliations:** 1 Organization for the Strategic Coordination of Research and Intellectual Property, Meiji University, Kanagawa 214-8571, Japan; saishu@meiji.ac.jp (S.Y.); tf00002@isc.meiji.ac.jp (T.K.); 2 Institute of Reproduction and Endocrinology, Meiji University, Kanagawa 214-8571, Japan; 3 Division of Life Science, Graduate School of Agriculture, Meiji University, Kanagawa 214-8571, Japan; 4 Department of Life Science, School of Agriculture, Meiji University, Kanagawa 214-8571, Japan

**Keywords:** pituitary, stem/progenitor cells, niche, signaling molecules, cell regeneration

## Abstract

The anterior lobe of the pituitary gland is a master endocrine tissue composed of five types of endocrine cells. Although the turnover rate of pituitary endocrine cells is as low as about 1.6% per day, recent studies have demonstrated that Sex-determining region Y-box 2 (SOX2)^+^-cells exist as pituitary stem/progenitor cells in the adult anterior lobe and contribute to cell regeneration. Notably, SOX2^+^-pituitary stem/progenitor cells form two types of niches in this tissue: the marginal cell layer (MCL-niche) and the dense cell clusters scattering in the parenchyma (parenchymal-niche). However, little is known about the mechanisms and factors for regulating the pituitary stem/progenitor cell niches, as well as the functional differences between the two types of niches. Elucidation of the regulatory mechanisms in the niches might enable us to understand the cell regeneration system that acts in accordance with physiological demands in the adult pituitary. In this review, so as to reveal the regulatory mechanisms of the two types of niche, we summarize the regulatory factors and their roles in the adult rodent pituitary niches by focusing on three components: soluble factors, cell surface proteins and extracellular matrixes.

## 1. Introduction

The pituitary gland is known as a key endocrine tissue producing various hormones to regulate homeostasis. This gland is embryologically and anatomically composed of two different entities: the adenohypophysis (anterior pituitary) composed of the anterior and intermediate lobes, and the neurohypophysis of the posterior lobe [1]. While in human the intermediate lobe fuses with the anterior lobe soon after birth and exists as a rudiment, the intermediate lobe definitely exists in rodents [2]. In particular, the anterior lobe has five types of endocrine cells: somatotrophs producing growth hormone (GH), mammotrophs producing prolactin (PRL), thyrotrophs producing thyroid-stimulating hormone (TSH), gonadotrophs producing luteinizing hormone (LH)- and follicle-stimulating hormone (FSH), along with corticotrophs producing adrenocorticotrophic hormone (ACTH) [1]. Besides these endocrine cells, stem cells have been assumed to exist from 1969 [3], since the number of specific types of endocrine cells increases mainly by proliferation of undifferentiated cells in physiological states such as pregnancy and extirpation of target organs [4,5]. For about 10 years, the pituitary stem/progenitor cells have been identified by several approaches such as side-population (SP) assay, sphere-forming assay and gene-tracing analyses, as well as identification of the factors expressed therein. Collectively, *in vitro* and *in vivo* studies concluded that high-mobility group (HMG) box transcription factor, Sex-determining region Y-box 2 (SOX2)-positive cells (SOX2^+^-cells) exist as the pituitary stem/progenitor cells in the rodent anterior lobe during both the embryonic and postnatal periods [6,7,8].

For the important issue of maintaining stemness, niches, which are a micro-environment specialized for maintaining stem cells were noted and identified in various tissues, such as bone marrow [9], the crypt in the intestine [10,11], the subventricular zone (SVZ) in the brain [12] and hair follicles in the skin [13]. Accumulating studies have demonstrated that these niches regulate the multipotency, self-renewal, asymmetric cell division and migration from niches for differentiation via signaling from soluble factors [11], cell surface proteins [14] and extracellular matrices (ECMs) [15].

In the adult rodent pituitary, the localization pattern of SOX2^+^-cells suggested that the anterior lobe of pituitary has two types of stem/progenitor cell niche; one is the marginal cell layer (MCL-niche) and the other is the SOX2^+^-cell clusters scattering in the parenchyma of the anterior lobe (parenchymal-niche). However, little is known about the mechanisms and factors regulating pituitary stem/progenitor cell niches, nor about the functional differences between the two types of pituitary niches.

In this review, we follow up about the regulatory factors of the adult rodent pituitary stem/progenitor cell niches, focusing on their signaling with soluble factors, cell surface proteins and ECMs.

## 2. Pituitary Stem/Progenitor Cells and Their Niches

### 2.1. Identification of Pituitary Stem/Progenitor Cells

#### 2.1.1. Side-Population Cells

The first convincing report about adult pituitary stem/progenitor cells was the separation and analysis of side-population (SP) cells reported by Vankelecom and colleagues [16]. The SP cell is known as a stem cell population enriched from dispersed cells by a difference in the efflux capacity for the dye Hoechst 33,342 using flow-cytometry [17]. About 1.5% of the cells in the anterior lobe of the pituitary of 3- to 8-week-old mice were recovered as SP [16,18]. These SP cells were furthermore separated into two fractions by the level of *Sca1* (stem cell antigen-1)-expression: *Sca1*^high^-SP (showing high *Sca1*-expression, about 60% of SP) and non-*Sca1*^high^-SP (showing low *Sca1*-expression, about 40% of SP). Microarray and semi-qPCR analyses demonstrated that pituitary stem/progenitor factors, *Sox2* and *Sox9* (described in Section 2.1.2.), and stem cell related-genes, *Lgr5*, *CD44* and *Nanog*, are enriched in non-*Sca1*^high^-SP when compared to *Sca1*^high^-SP (composed mostly of endothelial phenotype cells) and the main population (MP, composed mostly of endocrine cells). Moreover, early embryonic transcription factors such as *Lhx4*, *Prop1*, *Pax6* and *Hey1* [1] were also enriched in non-*Sca1*^high^-SP. In relation to the characteristics of stem cells, non-*Sca1*^high^-SP clearly showed a sphere-forming ability, indicating a potential for self-renewal similar to neuro-, mammo- and prostate-spheres [19]. Notably, immunostaining demonstrated that these pituispheres (pituitary derived sphere) were negative for any hormones, indicating that they are composed of undifferentiated cells [16,20]. These data suggested that pituitary stem/progenitor cells having the ability to self-renew hide in the non-endocrine cells in the adult pituitary.

#### 2.1.2. SOX2^+^-Cells

A few years after their reports about pituitary SP, Fauquier *et al.* identified SOX2^+^-cells as non-endocrine cells [7]. Immunohistochemistry demonstrated that SOX2^+^-cells initially present in all cells of the pituitary primordium, Rathke’s pouch. During pituitary development, although the number of SOX2^+^-cells decreases, they are continuously present in the adult pituitary of the mouse [7] and rat [21]. Notably, Fauquier *et al.* showed that SOX2^+^-cells have the ability to form spheres and differentiate into all types of endocrine cells *in vitro* [7]. More recently, two different research groups simultaneously reported evidence that SOX2^+^-cells supply endocrine cells *in vivo*, using gene-tracing analysis by temporal tamoxifen-induction of transgenic mouse [6,8]. Andoniadou *et al.* [6] and Rizzoti *et al.* [8] demonstrated that SOX2^+^-cells certainly self-renew and supply all types of endocrine cells in both the embryonic and adult pituitaries using *Sox2^CreERT2/+^*; *R26^YFP/+^* mice, which are generated by crossing *Sox2-*CreERT2 and ROSA26-flox-stop-YFP mice. Interestingly, Andoniadou *et al.* [6] also showed that the turnover rate of pituitary cells is comparatively slower than that of other tissues, and that pituitary stem/progenitor cells are non-short-lived ones under normal physiological conditions, since only about 30% of differentiated cells are derived from YFP-labeled SOX2^+^-cells which are negative for hormones even after year-long tracing. Rizzoti *et al.*, using *Sox9^Ires−CreERT2/+^*; *R26^YFP/+^* mice in addition to *Sox2^CreERT2/+^*; *R26^YFP/+^* mice, further demonstrated that about 20% of newly generated ACTH-cells in acute adrenalectomy are derived from SOX9^+^-cells, which are a main-population (about 98%) of SOX2^+^-cells in the anterior lobe [8].

#### 2.1.3. Calcium-Binding Protein B (S100β^+^)-Cells

Another interesting cell population is calcium-binding protein B (S100β)^+^-cells [22]. S100β^+^-cells have been regarded as typical non-endocrine cells, and first appear in the anterior pituitary after birth [23]. They form cell-networks via their long processes, and produce numerous growth factors such as activators of stem cell proliferation (*i.e.*, basic fibroblast growth factor, bFGF; epidermal growth factor, EGF; and leukemia inhibitory factor, LIF) [24], IL-6 [25] and angiogenic factors (e.g., vascular endothelial growth factor [26]), in addition to several receptors such as β1- and β2-adrenergic receptors [27], angiotensin II receptor-1 [28], pituitary adenylate cyclase-activating peptide (PACAP) receptors [29] and TSH receptor [30]. Interestingly, S100β^+^-cells show multi-functions as phagocytes [31], supportive cells [22], cells forming a cell-network via gap junctions [32], and as cells regulating hormone release [33]. Notably, about 85% of S100β^+^-cells are composed of SOX2^+^-cells in the adult rat pituitary [21], and some of them show an ability to differentiate into endocrine cells [34]. Therefore, a sub-population of S100β^+^-cells is regarded to be adult pituitary stem/progenitor cells. However, functional differences among SOX2^+^-stem/progenitor cells with and without expression of S100β are not yet clarified.

### 2.2. Construction of the Two Types of Pituitary Stem/Progenitor Cell Niche

#### 2.2.1. Stem/Progenitor Cell Niche

**Figure 1:**
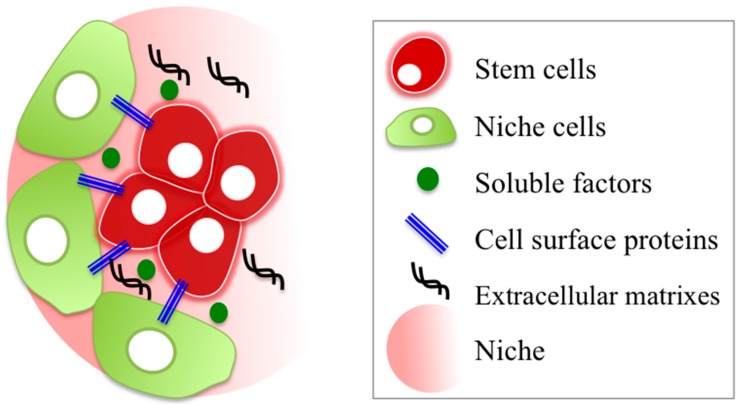
Schematic representation of a microenvironment ”niche.” A niche is typically constructed by stem cells and niche cells. Interaction between stem cells and niche cells via soluble factors, cell surface proteins and extracellular matrixes (ECMs) regulates the stemness and differentiation.

Adult stem cells are known to present in a microenvironment “niche”, providing architectural support and molecular signals for regulating quiescence, self-renewal and differentiation for the maintenance of various tissues. A typical niche is constructed by stem cells and “niche cells” which regulate stem cell functions via signaling with soluble factors, cell surface proteins and ECMs (Figure 1), such as the hub cells in the *Drosophila* testis [35], Paneth cells in the crypt [36] and ependymal cells in the SVZ [37].

#### 2.2.2. Two Types of Niche Constructed in the Adult Pituitary

In the pituitary, an analysis conducted for the localization of SOX2^+^-cells has revealed the presence of stem/progenitor cell niches in both embryonic and adult pituitaries [38,39]. During early pituitary development (rat E12.5 to E13.5), all cells in Rathke’s pouch are SOX2^+^-cells [7,40]. However, during late to neonatal pituitary development, SOX2^+^-cells gradually decrease the cell number and are densely located in the MCL facing the residual lumen [7,40].

**Figure 2:**
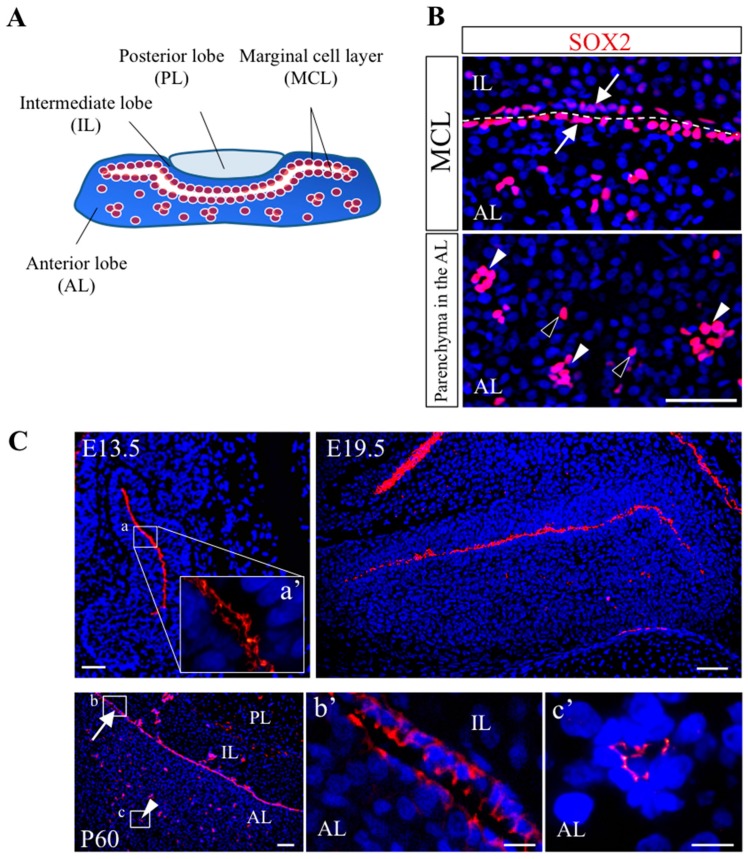
Localization of two types of stem/progenitor cell niche in the adult rat pituitary. (**A**) Schematic representation of the pituitary gland and localization of SOX2^+^-stem/progenitor cells. The pituitary gland is composed of three lobes: anterior lobe (AL), intermediate lobe (IL) and posterior lobe (PL); (**B**) Immunohistochemistry for SOX2. Pituitary stem/progenitor cells are visualized with transcription factor SOX2 (red). Sections in the coronal plane were prepared from the adult rat pituitaries (postnatal day 60). Scale bar: 50 µm. The single cell layer facing the residual lumen is the marginal cell layer (MCL). Especially in the anterior lobe, SOX2^+^-stem/progenitor cells show three localization patterns: lining the MCL (MCL-niche: arrows), forming clusters (parenchymal-niche: closed arrowheads) and singly scattering in the parenchyma (open arrowheads); (**C**) Immunohistochemistry for coxsackievirus and adenovirus receptor (CAR) (red) as an index of the stem/progenitor cell niche. Although CAR exists in the MCL throughout life, CAR starts to appear in the clusters of parenchyma after birth. Boxed area (**a**) in E13.5 is enlarged (**a’**). The MCL (arrow) and cluster (closed arrowhead) in boxed area (**b**,**c**) are enlarged (**b’**,**c’**), respectively. The localization of CAR in the MCL-niche (arrow) and the dense clusters in the parenchymal-niche (closed arrowhead) are defined as “primary” and “secondary” niches, respectively. Scale bar: 50 µm. Panel **C** is reproduced and modified from reference [39], with permission © 2013, Springer.

On the other hand, during postnatal periods, SOX2^+^-cells localize by forming dense clusters scattering in the parenchyma of anterior lobes (Figure 2B, closed arrowheads) in addition to the MCL (Figure 2B, arrows). Therefore, in the adult pituitary, three localization patterns of SOX2^+^-cells exist: lining the MCL (Figure 2B, arrows), clustering in the parenchyma (Figure 2B, closed arrowheads), and singly scattering in the parenchyma (Figure 2B, open arrowheads).

Chen *et al.* demonstrated by immunohistochemistry that the rat pituitary stem/progenitor cell niche is structured by the homophilic tight-junction forming factor CAR (Coxsackievirus and adenovirus receptor) [39], which is expressed in ependymal cell niches in the SVZ in the brain [41], as well as niches in the intestine, hair follicle and liver regardless of the origin of germ layers (data not shown). In the anterior pituitary, CAR localized only in the apical side of the MCL (Figure 2C, E13.5) throughout life (Figure 2C, E19.5) [39]. Furthermore, CAR localized only in the apical membrane of SOX2^+^-cells forming clusters in the parenchyma, which appeared after birth, in the adult anterior lobe (Figure 2C, P60). These SOX2^+^/CAR^+^-clusters appeared after birth and rapidly increased their number during the postnatal growth wave (first to second weeks after birth) [39]. Collectively, two types of niche, the MCL (MCL-niche) and clusters in the parenchyma (parenchymal-niche), which are maintained by tight junction, exist in the adult rat pituitary [39]. The MCL-niche and parenchymal-niche are regarded as “primary” and “secondary” pituitary niches, respectively [42,43]. However, niche cells have not been identified in both pituitary niches.

While SOX2^+^-pituitary stem/progenitor cells are kept in their niches in the adult pituitary, they have to launch from these niches for differentiation and cell regeneration. To migrate from the niche, they have to change their properties by epithelial-mesenchymal transition (EMT) and break the tight junctions constructed via cell adhesion molecules (e.g., E-cadherin [7] and CAR [39]). Recent finding that several key factors such as *Snail1*, *Twist1/2* and *Zeb*1/*2* were enriched in non-*Sca1*^high^-SP have demonstrated the potential abilities to initiate EMT in pituitary stem/progenitor cells (see [42] in particular Table 1).

Although two types of niche have been collectively identified in the adult pituitary, the functional differences between them as well as the presence of niche cells remain to be elucidated. To understand these issues in addition to the mechanisms of cell regeneration in the pituitary niches, it is important to investigate the factors regulating the ability to self-renew, maintain stemness and induce cell migration

**Table 1:** Candidate factors for regulating the stem/progenitor cell niches in the adult pituitary.

Signaling Types	Gene Symbol	Gene Title	Description	Localization and/or Expression in the							References
				MCL-AL	MCL-IL	SOX2^+^-Cell Clusters	Non-SOX2^+^-Cell	S100β^+^-Cell	SP	Analyzed Species	
Soluble factor signaling	*Fgfr*	basic fibroblast growth factor receptor	Receptor for growth factor	–	–	–	–	–	c	m	[20]
	*Egfr*	epidermal growth factor receptor	Receptor for growth factor	–	–	–	–	–	c	m	[20]
	*Lifr*	leukemia inhibitory factor receptor	Receptor for growth factor	–	–	–	–	–	c	m	[20]
	*Ntn*	Neurturin	Growth factor	–	–	–	a	–	–	r, h	[44]
	*Gfra2*	GDNF receptor α 2	Receptor for growth factor	a	a	–	–	–	–	m, r, h	[44]
	*Cxcl12* (*Sdf1*)	Stromal cell-derived factor-1	Chemokine	–	–	–	–	a, b, c, d	c	m, r	[42,45]
	*Cxcr4*	C-X-C chemokine receptor type 4	Receptor for Chemokine	–	–	–	a, b, c, d	a, b, c, d	c	m, r	[20,45,46]
Juxtacrine signaling	*Notch1*	Notch receptor 1	Receptor for Notch signaling	b	b	b	–	b, c	c	m, r	[18,20,47]
	*Notch2*	Notch receptor 2	Receptor for Notch signaling	b	b	b	–	b, c	c	m, r	[18,20,47]
	*Notch3*	Notch receptor 3	Receptor for Notch signaling	–	–	–	–	–	c	m	[18,20]
	*Notch4*	Notch receptor 4	Receptor for Notch signaling	–	–	–	–	–	c	m	[18,20]
	*Jag1*	Jagged1	Ligand for Notch signaling	b	b	b	–	b, c	c	m, r	[20,47]
	*Jag2*	Jagged2	Ligand for Notch signaling	–	b	–	b (in the IL)	–	–	r	[47]
	*Dll4*	Delta-like protein 4	Ligand for Notch signaling	–	–	–	–	–	c	m	[20]
	*Efn-B2*	Ephrin-B2	Ligand for ephrin/Eph signaling	a	a	a	–	a	c	m, r	[42,48]
ECM-to-cell signaling	*Itga1*	Integrin, α1	Linkage of the ECM to the cells	–	–	–	c	c	–	r	[49]
	*Itga3*	Integrin, α3	Linkage of the ECM to the cells	–	–	–	c	c	–	r	[49]
	*Itga6*	Integrin, α6	Linkage of the ECM to the cells	–	–	–	c	c	–	r	[49]
	*Itgb1*	Integrin, β1	Linkage of the ECM to the cells	–	–	–	c	a, c	–	r	[49]
	*Lama5*	Laminin, α5	ECM	b	b	b	–	–	–	r	[50]
	*Sdc4*	Syndecan 4	Transmembrane proteoglycan	–	–	–	c	c, d	–	r	[51]
	*Dcn*	Decorin	SLRPs	–	–	–	–	b	–	r	[52]
	*Bgn*	Biglycan	SLRPs	–	–	–	–	b	–	r	[52]
	*Fmod*	Fibromodulin	SLRPs	–	–	–	–	b	–	r	[52]
	*Lum*	Lumican	SLRPs	–	–	–	–	b	–	r	[52]
	*Prelp*	Proline/arginine-rich end leucine-rich repeat protein	SLRPs	–	–	–	–	b	–	r	[52]
	*Ogn*	Osteoglycan	SLRPs	–	–	–	–	b	–	r	[52]

Each localization and/or expression is demonstrated by immunohistochemistry (a), in situ hybridization (b), (Semi-) qPCR (c) and Western blotting (d); A ”SP” column indicates the genes enriched in non-*Sca1*^high^-SP (side-population) than main population ones; In “Analyzed species” column, m, r and h indicate mouse, rat and human, respectively; MCL-AL, the marginal cell layer in the anterior lobe; MCL-IL, the marginal cell layer in the intermediate lobe; ECM, extracellular matrix; GDNF, glial cell-line derived neurotrophic factor; SLRPs, small leucine-rich proteoglycans.

## 3. Candidates for Regulator of Pituitary Stem/Progenitor Cell Niches

One of the well-characterized niches in mammals is the crypt in the small intestine. The components of niche are stem cells expressing *Lgr5* (crypt base columnar cell) [53] and *Bmi1* (+4 cell) [54], and Paneth cells functioning as niche cells, which come in close contact with stem cells [11]. These stem cells are kept in the bottom of the crypt and their stemness are maintained by interaction with Paneth cells via soluble factors (Wnt ligand [55] and EGF [56]), juxtacrine factors (Notch signaling [57] and ephrin/Eph signaling [58]) and ECMs [11,14,59]. These regulatory factors characterized in the crypt are found in other stem/progenitor cell niches including pituitary stem/progenitor cells. As described in Section 2.2., two types of the pituitary stem/progenitor cell niches have been identified but differences in their regulatory mechanisms are still obscure. In this section, we describe the candidate factors and systems for regulating stem/progenitor cells in the adult pituitary, focusing on three signaling systems via soluble factors, cell surface factors (juxtacrine factors) and ECMs (Table 1).

### 3.1. Soluble Factor Signaling

#### 3.1.1. Growth Factor Signaling

Growth factors are the most-investigated factors, having a regulatory component in the stem/progenitor cell niche in various tissues. In the adult mouse pituitary, Chen *et al.* found several candidates for regulatory factors using aggregated cell culture and side-population cells from the adult anterior lobe [18]. Chen *et al.* focused on growth factors produced in the adult pituitary such as bFGF [24], EGF [60], LIF [61] and nerve growth factor (NGF) [62]. They treated each factor against aggregated cells of the adult mouse pituitary cultured for 10–14 days, followed by fractionation of SP. Proliferation and expansion of SP were observed by bFGF-, EGF- and LIF-treatment, whereas NGF-treatment showed no effect [18]. Corresponding to these results, *Fgfr-*, *Egfr-* and *Lifr-*transcripts were enriched in non-*Sca1*^high^-SP (Table 1) [20]. Interestingly, these factors are commonly produced by non-hormonal S100β^+^-cells, and are also known to affect endocrine cells [63]. These data suggest that bFGF, EGF and LIF produced by S100β^+^-cells contribute to regulate pituitary stem/progenitor cells as well as endocrine cells. Notably, bFGF and EGF are well-known to be essential for the proliferation of stem/progenitor cells in the mouse pituitary embryogenesis [1]. Therefore, a part of the growth factor signalings in the embryonic pituitary continuously participates in the adult pituitary for maintaining stem/progenitor cells.

#### 3.1.2. Neurturin/Glial Cell-Line Derived Neurotrophic Factor (GDNF) Receptorα2 (GFRα2)/Co-Receptor of the Tyrosine Kinase (RET) Signaling

Another interesting factor is the glial cell-line derived neurotrophic factor (GDNF). GDNF family in mammals is composed of four factors: GDNF, Neurturin (NRTN), Persephin (PSPN) and Artemin (ARTN). They bind to GDNF receptor α (GFRα1-4), which acts as a co-receptor of the tyrosine kinase (RET) [64]. GDNF-GFRα-RET signaling is involved in cell proliferation and migration of germline stem cells [65] and neuronal cells [66]. Garcia-Lavandeira *et al.* [44] reported that in the pituitary, GFRα2, which is a specific NRTN receptor, is expressed in about 0.9% of the cells in the adult mouse pituitary, and that more than 90% and 50% of GFRα2^+^-cells are positive for SOX2/SOX9 and S100β, respectively. Interestingly, GFRα2^+^-cells are detected in the MCL but not in the parenchymal niches (Table 1).

Isolation of GFRα2^+^-cells using anti-GFRα2 antibody coupled to magnetic beads and following sphere forming assay demonstrated that only GFRα2^+^-cells have the ability to form pituispheres and differentiate into all types of endocrine cells. Notably, treatment with NRTN, a specific ligand of GFRα2, promoted the sphere-forming efficiency of GFRα2^+^-cells. In contrast to the localization of GFRα2 in the MCL, NRTN is produced in an exclusively small number of cells dispersed over the anterior lobe, except in the MCL of human and rat pituitary (Table 1). From these data, Garcia-Lavandeira *et al.* hypothesized that gradient signaling of NRTN-GFRα2-RET may act as regulatory signaling in the MCL-niche to promote proliferation and/or migration [44]. However, no direct evidence has yet been provided.

#### 3.1.3. CXCL12/CXCR4 Signaling

Understanding the mechanisms involved in migration from niche to differentiation is an important issue. Especially, CXC chemokine and their receptor signaling introduced by CXCL12 (same as SDF1, stromal cell-derived factor-1) and its receptor CXCR4, are known to promote the migration of neural stem cells [67], primordial germ cells [65], cancer stem cells [68] and neural crest cells [69], and are involved in the homing and maintenance of hematopoietic stem cells (HSCs) [70,71,72]. As the first report about CXCL12/CXCR4-axis in the pituitary stem/progenitor cells, Vankelecom and his colleagues showed that both *Cxcl12* and *Cxcr4* are enriched in mouse non-*Sca1*^high^-SP (Table 1) [20] (reviewed in [42]).

A few years later, Horiguchi *et al.* demonstrated that *Cxcl12* is specifically expressed in rat S100β^+^-cells (Table 1) [45]. In contrast, its receptor *Cxcr4* was expressed in both S100β-positive and -negative cells (including at least GH^+^-cells [46]) (Table 1). An *in vitro* culture system of S100β^+^-cells demonstrated that activation of CXCL12/CXCR4 signaling by CXCL12-treatment promotes cell migration, invasion and interconnection of S100β^+^-cells [45]. More recently, Horiguchi *et al.* demonstrated that SLUG, one of the key transcription factors for EMT, exists in about 80% and 55% of S100β^+^-cells in the rat pituitary at postnatal days (P) 10 and P60, respectively, and up-regulates *Cxcl12* expression [73]. Taken together with the fact that 85% of S100β^+^-cells are composed of SOX2^+^-stem/progenitor cells in the rat anterior lobe (described in Section 2.1.) [21], these data suggest that CXCL12/CXCR4 signaling plays a role in EMT and migration of pituitary stem/progenitor cells in the adult rat pituitary by a paracrine and/or autocrine system via networks of S100β^+^-cells. Further studies concerning this signaling focused on the MCL and parenchymal niches may well provide valuable information about the stem/progenitor cells’ mechanism of migration from niche for differentiation.

### 3.2. Cell Surface Factor Signaling

Cell surface factor signaling is also known as cell-to-cell contact-dependent signaling since a ligand (e.g., protein, oligosaccharide and lipid) presents on a cell membrane as well as its receptor. This signaling has important roles in the cell communication regulating cell migration, boundary formation and differentiation during organogenesis, in addition to the various stem cell niches. Furthermore, clarification of their cell surface localization might enable us to identify the cells regulating stem cells, such as a niche cell. In this section, we summarize the two cell surface factor signaling molecules (also called juxtacrine factor), Notch and ephrin/Eph, in the pituitary stem/progenitor cell niches.

#### 3.2.1. Notch and Its Ligand

Notch signaling is one of the most-investigated types of juxtacrine signaling, and is activated by cell-to-cell interaction between each of the cells producing ligands (*Dll1*, *Dll3*, *Dll4*, *Jagged1* and *Jagged2*) and receptors (*Notch1-4*). NOTCH is a transmembrane protein composed of extracellular (NECD), transmembrane, and intracellular (NICD) domains. Interaction with NOTCH and its ligand promotes cleavage of NICD, which translocates into the nucleus, resulting in transcriptional activation by forming a complex with Mastermind-like protein (MAMLs) and recombination signal binding protein for immunoglobulin kappa J region (RBP-J) [74]. Notch signaling has an important role not only in development, but also in maintenance of stem/progenitor cells by regulating proliferation, asymmetric cell division and differentiation, in various niches such as the crypt, SVZ, hair follicle and bone marrow [74]. Also in pituitary development, activation of Notch signaling is required for maintenance of the proliferative state of progenitor cells which give rise to multiple cell lineages [75]. More recently, ablation of the Notch signaling in the embryonic pituitary stem/progenitor cells using conditional knockout mice (*Rbp-j*^fl/fl^*Prop1-Cre*) demonstrated that the number of SOX2^+^-cells is drastically decreased in the postnatal pituitary [76].

Vankelecom and colleagues reported that in the adult mouse pituitary, key factors of the Notch signaling pathway (e.g., *Jagged1*, *Notch2*, *Notch3*, *Hes1* and *Hey1*) are enriched in non-*Sca1*^high^-SP (Table 1) [20,42]. More recently, Tando *et al.* reported the localization of the receptors (*Notch1*, *Notch2*, *Notch3* and *Notch4*) and ligands (*Jagged1*, *Jagged2*, *Dll1*, *Dll3* and *Dll4*) in the adult rat pituitary by *in situ* hybridization [47]. Among them, *Notch1*, *Notch2*, *Jagged1* and *Jagged2* existed in almost 50% of cells in the MCL, and *Notch1*, *Notch2* and *Jagged1* were also found in the parenchymal-niche as S100^+^-cells (Table 1). These reports suggested that Notch signaling plays a role in both types of pituitary niches.

Functional analysis of Notch signaling in the postnatal mouse pituitary stem/progenitor cells was also reported [77]. Inhibition of Notch signaling in the early embryonic pituitary using conditional knockout (*Notch2*^+/fl^*Foxg1*^+/cre^) decreased the number of SOX2^+^ and SOX9^+^-cells in both the MCL and parenchymal-niches, along with the proliferating cells in the early postnatal pituitary. In addition, *in vivo* and *in vitro* treatment of *N*-[*N*-(3,5-difluorophenacetyl-l-alanyl)]-*S*-phenylglycine *t*-butyl ester (DAPT), an inhibitor of Notch signaling, decreased the number of proliferating cells in the early postnatal pituitary. Furthermore, DAPT-treatment inhibited the extensive effect of bFGF on the proportion of SP [18]. The proliferation of S100β^+^-cells was decreased by DAPT-treatment, but was increased by treatment with soluble-Jagged1 [47]. These data suggest that Notch signaling activates the proliferation of stem/progenitor cells to maintain progenitor population in the postnatal pituitary.

#### 3.2.2. Ephrin and Eph

Two other interesting juxtacrine factors are ephrins and Ephs. Ephs belong to the subfamily of receptor tyrosine kinases and are composed of two subclasses, Eph A with nine members and Eph B with five members [78,79]. Their ligands, ephrins (Eph receptor interacting proteins), are cell-surface associated proteins composed of two subclasses, ephrin-A with five members and ephrin-B with three members, based on their structure and function [78,79]. While ephrin-As typically bind to EphAs, and ephrin-Bs bind to EphBs, there are a few exceptions, such as the interaction of ephrin-B2 and -B3 with EphA4 [80]. Their interaction triggers bidirectional (forward and reverse) signaling which plays a role in boundary formation by regulating cell repulsion and migration into many tissues [49,81,82]. For instance, involvement of ephrin-Bs signaling in the cell attachment and migration is known to take place in ovary, glioma, melanoma and intestinal epithelia-derived cell lines [83,84,85]. Recent studies revealed that ephrin/Eph signaling is crucial in maintaining stemness and keeping stem cells in their niches such as the crypt [58], subgranular zone [86] and subventricular zone [37].

In the mouse pituitary, *ephrin-B2*, *ephrin-B3*, *EphB1*, *EphB2* and *EphB3* were enriched in non-*Sca1*^high^-SP as compared to those in *Sca1*^high^-SP and/or MP (Table 1) [42]. More recently, our group demonstrated by immunohistochemistry that one of the ligands, ephrin-B2, is specifically localized in cells positive for SOX2, E-cadherin, S100β and CAR, but negative for hormones in both the MCL- and parenchymal-niches of the adult rat pituitary (Table 1) [48]. Notably, during the early postnatal periods when stem/progenitor cells are assumed to migrate from the MCL to parenchyma [39], ephrin-B2^+^-cells formed multiple cell layers beneath the MCL, changing their cellular localization to basolateral cell membranes. Taken together with reports that ephrin-Bs signaling regulates cell attachment and migration [83,84,85], ephrin-B2 may promote cell migration from the pituitary niche for differentiation. However, no evidence as yet has been reported to confirm this idea, nor the identification and localization of its partner molecule, Ephs.

To summarize, in the pituitary, Notch and ephrin/Eph molecules are distinctively expressed in both types of pituitary niche, and may well contribute to maintaining stem/progenitor cell populations. From another standpoint regarding the features of the juxtacrine factor, the localization of both Notch and their ligands seems to be expressed in stem/progenitor cells. Furthermore, our study recently demonstrated that EphB3, one of the partners of ephrin-B2, is expressed in the same SOX2^+^-cells of the both pituitary niches (data not shown). These data led us to hypothesize that pituitary stem/progenitor niches do not have definite niche cells such as Paneth cells in the crypt and ependymal cells in the SVZ, and are constructed by multiple stem/progenitor cell populations which regulate themselves by close intercellular communication. Further studies exploring the localization of juxtacrine factors by immunostaining and functional analyses might reveal whether niche cells are present or absent, in addition to revealing the functions of Notch and ephrin/Eph in the pituitary stem/progenitor cell niches.

### 3.3. Extracellular Matrixes (ECMs)

#### 3.3.1. ECMs and Integrins

ECMs play important roles in the formation of the basement membrane, regulating the presentation of soluble factors (matricrine factors) as well as the construction of supportive scaffolds. In the stem cell niches, ECMs provide a specialized microenvironment to regulate maintenance, self-renewal and differentiation of stem cells [15].

Major components of ECMs in mammals are laminin, collagen, fibronectin and proteoglycan. Among them, laminin is one of the components of the basement membrane [87]. Laminin is a heterotrimeric glycoprotein composed of α, β and γ chains, and has 19 isoforms [87]. The cell-to-ECM adhesion is regulated by more than 20 cell surface receptors such as integrin, transmembrane proteoglycan, dystroglycan and the immunoglobulin superfamily. In particular, integrin is the most investigated factor and forms seven heterodimers with an α- and a β-subunit [88]. Recent studies have reported that several ECM-receptors are produced by the stem cells [89]; for instance, α6 and β1 integrin are expressed in both embryonic and adult neural stem cells [90,91,92].

Few experiments with ECMs focusing on the pituitary stem/progenitor cell niches have been conducted. However, growth-factor reduced Matrigel, which is a mixture of ECM proteins (laminin, collagen IV, heparan sulfate proteoglycans and entactin/nidogen) produced by mouse sarcoma cell, induced the differentiation of pituisphere [7]. Therefore, there is little doubt that ECMs are involved in the regulation of stemness and differentiation of pituitary stem/progenitor cells.

#### 3.3.2. ECMs in S100β^+^-Cells of the Pituitary

Horiguchi *et al.* reported that some components of ECMs are produced by S100β^+^-cells (Table 1) [49,52], making up to 85% of SOX2^+^-cells in the rat pituitary [21]. They isolated S100β^+^-cells using S100β-GFP TG rat [93] and analyzed their interaction with components of ECMs such as laminin, fibronectin and collagen type-I, -III and -IV by an *in vitro* culture system [49]. Cultivation of S100β^+^-cells on each ECM protein showed an extension of cytoplasmic processes and activation of proliferation, as well as formation of interconnections and gap junctions with neighboring S100β^+^-cells [49]. In particular, inhibition of integrin β1, which is one of the laminin receptors and a mediator of cell-to-ECM adhesion, suppressed the proliferation of S100β^+^-cells [94]. Taken together with the expression of *Integrin*-α*1*, *Integrin*-α*3*, *Integrin*-α*6* and *Integrin-*β*1* by S100β^+^-cells (Table 1) [49] and their ligand specificity [88], S100β^+^-cells might interact with laminin via integrin-α3β1and/or integrin-α6β1 in the pituitary [94].

In addition, the localizations of α, β and γ chains of laminin in the rat pituitary were also demonstrated by *in situ* hybridization [50,95]. Notably, *Laminin* α*5* mRNA was detected in Rathke’s pouch during rat E12.5 to E15.5. Moreover, in the postnatal pituitary, *Laminin* α*5* mRNA was found to exist in the MCL of both the anterior and intermediate lobes, in addition to the parenchymal-niche (Table 1) [50]. Laminin containing α5 (*i.e.*, α5β1γ1) is well-known to be expressed by pluripotent stem cells including embryonic stem (ES) cells and induced pluripotent stem (iPS) cells, and to maintain their pluripotency [96]. Collectively, laminin containing α5 chain could be involved in maintaining stem/progenitor cells in the pituitary niches.

Furthermore, Syndecan 4, which is a transmembrane proteoglycan that binds to ECM and soluble factors via their extracellular glycosaminoglycan chain, is expressed at a higher level by S100β^+^-cells, and leads to activation of α-actinin downstream of laminin (Table 1) [51]. In addition, small leucine-rich proteoglycans (SLRPs), which are a major family of proteoglycans such as *Decorin*, *Biglycan*, *Fibromodulin*, *Lumican*, *Proline/arginine-rich end leucine-rich repeat protein* (PRELP) and *Osteoglycan*, are expressed by some S100β^+^-cells and pericytes but not endocrine cells (Table 1) [52]. Although these reports focused on S100β^+^-cells, they led us to speculate that some of these ECMs are common to pituitary stem/progenitor cells, and might play important roles in constructing and controlling their niches via ECM-to-stem/progenitor cell interactions and recruitment of soluble factors.

## 4. Conclusions

SOX2^+^-cells in the anterior lobe show three localization patterns: the MCL and dense cell clusters in addition to cells not belonging to clusters in the parenchyma. Notably, the MCL and cell clusters in the parenchyma are regarded as the pituitary stem/progenitor niches. Recent studies focused on rodent pituitary SP cells and S100β^+^-cells have revealed the candidate factors for regulating stem/progenitor cells. However, the functional differences and contrasting regulatory systems between the two types of niche remain to be elucidated. In addition, the existence of pituitary niche cells has been unclear yet. Considering the morphological properties of S100β^+^-cells and the factors expressed by them, a part of S100β^+^-cells may be involved in sensing and responding to physiological states and in regulation of stem cell functions similar to niche cells. Further studies, perhaps by isolation of each niche and analysis of gene expression profiles, may well enable us to elucidate these issues, as well as answer the question, “How do stem/progenitor cells respond to physiological demand?”
